# Genome-Wide Screening Identified That miR-134 Acts as a Metastasis Suppressor by Targeting Integrin β1 in Hepatocellular Carcinoma

**DOI:** 10.1371/journal.pone.0087665

**Published:** 2014-02-03

**Authors:** Ruopeng Zha, Weijie Guo, Zhenfeng Zhang, Zhaoping Qiu, Qifeng Wang, Jie Ding, Shenglin Huang, Taoyang Chen, Jianren Gu, Ming Yao, Xianghuo He

**Affiliations:** 1 Shanghai Medical College, Fudan University, Shanghai, China; 2 State Key Laboratory of Oncogenes and Related Genes, Shanghai Cancer Institute, Renji Hospital, Shanghai Jiao Tong University School of Medicine, Shanghai, China; 3 Shanghai Cancer Hospital, Fudan University, Shanghai, China; 4 Qi Dong Liver Cancer Institute, Qi Dong, Jiangsu, China; University of North Carolina School of Medicine, United States of America

## Abstract

MicroRNAs (miRNAs) are small, single-stranded, non-coding RNAs that play pivotal roles in human cancer development and progression, such as tumor metastasis. Here, we identified the miRNAs that regulate hepatocellular carcinoma (HCC) cell migration by a high-throughput screening method using the classical wound-healing assay with time-lapse video microscopy and validation with a transwell migration assay. Eleven miRNAs (miR-134, -146b-3p, -188-3p, -525-3p, -661, -767-5p, -891a, -891b, -1244, -1247 and miR-1471) were found to promote or inhibit HCC cell migration. Further investigation revealed that miR-134 suppressed the invasion and metastasis of HCC cells *in vitro* and *in vivo*, and integrin beta 1 (ITGB1) was a direct and functional target gene of miR-134. Moreover, miR-134 inhibited the phosphorylation of focal adhesion kinase (FAK) and the activation of RhoA downstream of the ITGB1 pathway, thereby decreasing stress fiber formation and cell adhesion in HCC cells. In conclusion, we demonstrated that miR-134 is a novel metastasis suppressor in HCC and could be a potential therapeutic target for the treatment of HCC.

## Introduction

Worldwide, hepatocellular carcinoma (HCC) is the fifth most common malignancy in men, the seventh most common malignancy in women, and the third leading cause of cancer deaths, affecting over 500,000 people [1]. HCC usually develops as a consequence of underlying liver disease and is most often associated with cirrhosis. Additionally, metastasis or recurrence commonly causes mortality in patients [2]. Metastasis is a complex and multistep biological process that includes local invasion, intravasation, survival in the circulation, extravasation, arrest at a distant organ site, micrometastasis formation and metastatic colonization [3], [4]. Each step requires the coordinated spatial and temporal expression of genes and proteins. Many molecules contribute to this process, and some of these molecules are involved in the mechanical aspects, whereas others modulate regulatory pathways [3]. However, the molecular mechanism of metastasis has not been comprehensively elucidated.

MicroRNAs (miRNAs) are small, single-stranded, non-coding RNAs that post- transcriptionally regulate the expression of target genes, usually by binding to the 3′-UTR of the respective mRNAs [5]. They are involved in many physiological and pathological processes, such as embryonic development, cell differentiation, tumorigenesis and cancer metastasis [6], [7], [8], [9]. Many previous studies have demonstrated that miRNAs play important roles in cell migration and the metastasis of several tumor types, such as breast cancer, glioblastoma, colon cancer and hepatocellular carcinoma [10], [11], [12], [13], [14], [15], [16]. However, the function and mechanisms of miRNA-mediated regulation of HCC metastasis are largely unknown.

Here, we performed a large-scale screening to identify the miRNAs involved in HCC cell migration by classical wound-healing assays with time-lapse video microscopy. After independent validation with transwell migration assays, we found that 11 miRNAs regulated the migration of HCC cells *in vitro*. MicroRNA-134, the candidate tumor metastasis suppressor, is often downregulated in HCC [17] and significantly inhibited invasion and metastasis by targeting the ITGB1 pathway in HCC.

## Materials and Methods

### Ethics statement

All human materials were obtained with written informed consent, and the protocols were approved by the Ethical Review Committee of the World Health Organization of the Collaborating Center for Research in Human Production and authorized by the Shanghai Municipal Government. The samples used in this study also have been described in a previous study [18].

All experiments involving animals were performed in accordance with protocols approved by the Shanghai Medical Experimental Animal Care Commission.

### Oligonucleotides

All human miRNA mimics were obtained from GenePharma (Shanghai, China), and detailed information is listed in Table S1. The inhibitor (2′-*O*-methyl modification) of miR-134 was synthesized by Ribobio (Guangzhou, China).

### Cell culture and transfection

SK-HEP-1 and Huh-7 cells were purchased from ATCC, and the MHCC-LM3 cell line was a gift from Zhongshan Hospital, Shanghai, China. SK-HEP-1, Huh-7, and MHCC-LM3 cells were cultured in DMEM supplemented with 10% fetal bovine serum (Life Technologies, CA, USA), 100 IU/ml penicillin and 100 µg/ml streptomycin under a 5% CO_2_ atmosphere at 37°C. MicroRNA mimics were transfected using Lipofectamine RNAiMAX (Life Technologies) at a final concentration of 50 nM, and plasmid transfection was performed using Lipofectamine 2000 according to the manufacturer's instructions.

### Wound-healing assay combined with time-lapse video microscopy

For wound-healing assays, SK-HEP-1 cells were transfected with miRNA mimics for 48 hours and then plated at 2×10^5^ cells per well in 24-well plates with CytoSelect™ inserts (Cell Biolabs, INC. CA, USA). After 24 hours of incubation, the inserts were carefully removed from the wells, and the cells were washed once with growth medium and then imaged using a 10× objective on an Olympus IX81 motorized inverted microscope at 37°C under 5% CO_2_. Images of the wound-healing assays were acquired at 30-minute intervals for 24 hours. The percent closure of the cells into the wound field was measured using Image-Plus Pro software. The index of the inhibition of cell movement was calculated using the following equation: cell migration length in test miRNA condition/cell migration length in negative control condition×100%.

### *In vitro* migration and invasion assays

For the migration assay, 3×10^4^ SK-HEP-1 cells or 5×10^4^ Huh-7 cells were seeded in the top chamber of the insert (BD Biosciences, NJ, USA). For the invasion assay, 3×10^4^ SK-HEP-1 cells or 1×10^5^ Huh-7 cells were added to the upper chamber of the insert, which had previously been coated with 40 µl of Matrigel (BD Biosciences). After several hours of incubation at 37°C, cells that had migrated or invaded through the insert were fixed and stained in a dye solution containing 0.2% crystal violet and 20% methanol. The cells that had migrated or invaded through the insert were counted and imaged using an IX71 inverted microscope (Olympus Corp., Tokyo, Japan).

### Plasmid construction and lentiviral production

The sequence of pre-miR-134 and the open reading frame sequence of ITGB1 were amplified and cloned into the pWPXL lentiviral vector (a gift from Dr. Didier Trono) and the pLVX lentiviral vector (TaKaRa, Tokyo, Japan), respectively. The wild-type or mutant 3′-UTR of ITGB1 was inserted (Xho I+Not I) downstream of the stop codon of Renilla luciferase in the psiCHECK2 vector (Promega). Lentiviral production was performed according to instructions supplied by Addgene (www.addgene.org). SK-HEP-1, Huh-7 and LM3 cells were infected with recombinant lentivirus transducing units in the presence of 6 µg/ml polybrene (Sigma, MA, USA). The sequences of the primers used for PCR are listed in Table S2.

### RNA extraction and quantitative real-time PCR

Total RNA was extracted using TRIzol reagent (Invitrogen, CA, USA). Reverse-transcribed complementary DNA was synthesized using the PrimeScript RT reagent kit (TaKaRa). Real-time polymerase chain reaction (PCR) was performed using SYBR Premix ExTaq (TaKaRa). For miRNA detection, mature miR-134 was reverse-transcribed with specific stem-loop primers and quantified using a TaqMan probe. Normalization was performed with U6 or RNU6B small nuclear (Life Technologies). The sequences of the primers used for PCR are listed in Table S2.

### Human tissues

HCC liver tissues were collected from the surgical specimen cohort of the Qidong Liver Cancer Institute of Jiangsu Province and the first hospital of Zhejiang Province in China. HCC clinical information was collected from patient records, and the details are listed in Table S3.

### Cell proliferation and colony formation assays

Cell proliferation was measured by the Cell Counting Kit-8 (CCK-8) assay (Dojindo Corp., Kumamoto, Japan) according to the manufacturer's protocol. For colony formation assays, 5×10^2^ cells were plated into each well of a six-well plate and incubated at 37°C for 1 week. The cells were fixed and stained with 0.05% crystal violet in 20% methanol. Colonies were imaged and counted using an IX71 inverted microscope (Olympus Corp.). All samples were measured in triplicate, and three independent experiments were performed.

### Animal study and histology

For the *in vivo* orthotopic transplantation assay, 2×10^6^ MHCC-LM3 cells stably expressing miR-134 or infected with the control vector were suspended in 40 µl of DMEM/Matrigel (1∶1) for each mouse. Through an 8-mm transverse incision in the upper abdomen, each nude mouse (10 per group, male BALB/c-nude mice) was inoculated orthotopically in the left hepatic lobe using a microsyringe. After 8 weeks, mice were euthanized, and the livers were dissected and fixed in 10% formalin solution for standard histological examination.

### Microarray analysis

SK-HEP-1 cells were transfected with the miR-134 mimic or negative control for 48 hours. RNA was extracted for gene expression profiling on an Affymetrix Human U133 Plus 2.0 chip. Microarray analysis of mRNA expression profile was performed as previously described [19]. Genes with 0.5-fold change were designated as downregulated in miR-134-overexpressing cells compared with the negative control cells. The data were submitted into ArrayExpress (accession: E-MTAB-2018).

### Luciferase assay

SK-HEP-1 and Huh-7 cells were cultured in 96-well plates and cotransfected with 20 ng of psiCHECK2 reporter and 5 pmol miR-134 mimic or negative control. After 48 hours, Renilla luciferase activity was measured using the dual luciferase reporter assay system (Promega). Renilla luminescence units were normalized to *Firefly* luciferase luminescence units,

### Immunoblotting and Rho GTPase activation assays

Proteins were separated by SDS-PAGE and transferred to a nitrocellulose membrane (Bio-Rad, CA, USA). The membrane was blocked with 5% non-fat milk and incubated with a rabbit anti-ITGB1 antibody (mAb) (Cell Signaling Technology, MA, USA) (1∶1,000), rabbit anti-FAK antibody (mAb) (1∶1,000), rabbit anti-phospho-FAK antibody (Tyr397) (1∶1,000) or mouse anti-GAPDH antibody (mAb) (1∶2,000) (Thermo Fisher Scientific, MA, USA). The proteins were detected using enhanced chemiluminescence reagents (Thermo Fisher Scientific). Activation of Rho-family GTPase was detected using the Rho and Rac1/Cdc42 activation assay kit (Millipore Corp., MA, USA) according to the manufacturer's instructions.

### Immunofluorescence assay

SK-HEP-1 and Huh-7 cells were plated on glass slides and cultured for 48 hours. Cells were then fixed with 4% paraformaldehyde and permeabilized with 0.1% Triton-X 100, blocked in PBS containing 10% FBS for 1 hour at room temperature and incubated with a mouse monoclonal anti-α-tubulin (Sigma-Aldrich, MA, USA) or anti-vimentin (Dako, Glostrup, Denmark) antibodies overnight at 4°C. After three PBS washes, the slides were exposed to an Alexa Fluor® 488-conjugated secondary antibody (Life Technologies). F-actin and nuclei were stained with Alexa Fluor® 594 phalloidin and 4′, 6-diamidino-2-phenylindole dihydrochloride (DAPI), respectively. Cytoskeletal alterations were observed and documented using an Olympus FV1000 confocal microscope.

### Cell-matrix adhesion assay

Ninety-six-well plates were precoated with 20 mg/ml fibronectin (Sigma-Aldrich). SK-HEP-1 and Huh-7 cells were trypsinized and incubated in DMEM without serum for 45 or 30 minutes, respectively. The unattached cells were washed, and the attached cells were fixed with 70% ethanol and stained with 0.1% crystal violet in 20% ethanol. Then, the stained crystal violet was dissolved in 10% acetic acid, and the absorbance value (OD) was measured at 597 nm. Wells coated with bovine serum albumin served as a negative control, whereas cells that were incubated in complete culture medium for 8 hours served as a positive control. The cell matrix adhesion (CMA) index was calculated as the OD value (test-negative control)/OD value (positive control-negative control). All adhesion experiments were performed in triplicate wells and repeated at least three times.

### Statistical analysis

All results are presented as the mean ± SEM. The grouped data were analyzed by one-way ANOVA tests, and the differences between groups were assessed by the Bonferroni post-test following one-way ANOVA. The Mann-Whitney test was used for testing the difference between two group values. For tissue samples, differences between variables were assessed by the chi-square test or Fisher's exact test. P<0.05 was considered statistically significant.

## Results

### Large-scale screening for miRNAs that regulate cancer cell migration

**Figure 1:**
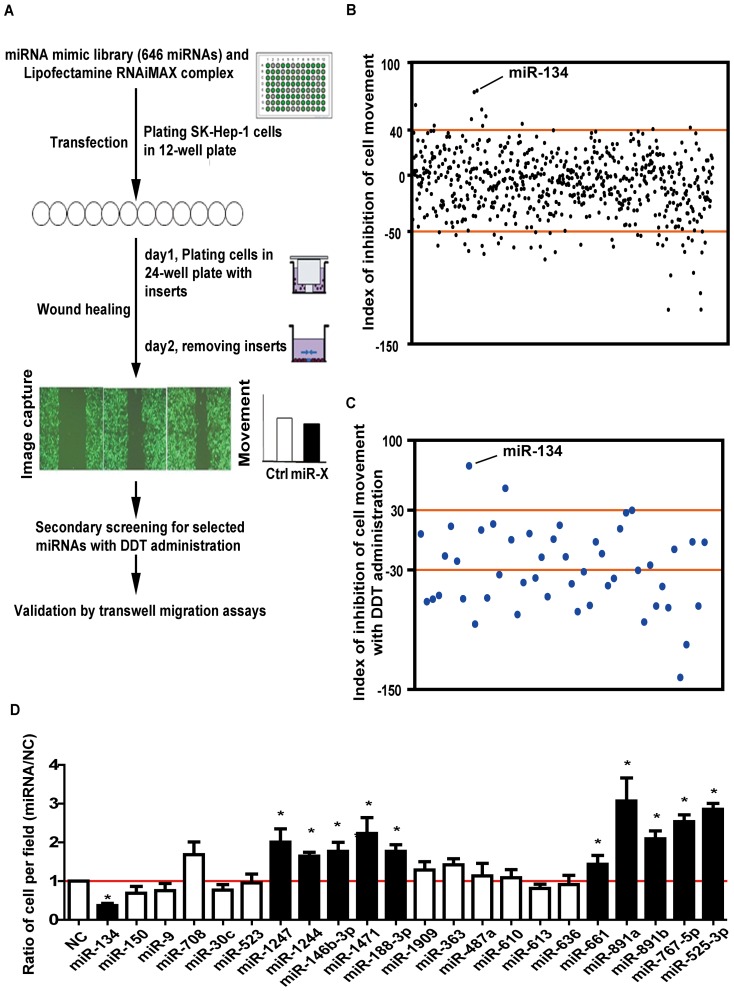
Large-scale screening for miRNAs that regulate cancer cell migration. **A.** Schematic diagram of the screening strategy. **B.** The results of the primary wound-healing screening. Each dot represents the inhibition index of an individual miRNA. The cutoff value was >40% (inhibition) or <−50% (promotion). **C.** The results of the second round of screening with administration of the proliferation inhibitor 2′, 3′- dideoxythymidine (DDT). **D.** Transwell migration assays for selected miRNAs (*p<0.05). SK-HEP-1 cells were plated into each well of a 12-well plate and transfected with individual miRNA mimics. The number of cells per field was counted after cells were placed into the top chamber of each insert for 5.5 hours. The results were normalized against data from cells transfected with the negative control. Differences between two groups were analyzed by the Mann-Whitney test.

To screen for the miRNAs regulating cell motility, we utilized time-lapse video microscopy in the wound-healing assay (Figure 1A). Representative videos of the wound region and cell morphology are shown in the Supplementary Videos (Video S1, Video S2 and Video S3). Cells transfected with miR-891a migrated more quickly into the wound region than the negative control cells. In total, 646 miRNAs (miRBase 15.0, Table S1) were investigated. The primary screening indicated that 48 miRNAs regulated cell migration with an index value under −50% or over 40% (Figure 1B and Table S4).

To eliminate the impact of cell multiplication, we repeated the wound-healing assay with the selected 48 miRNAs in the presence of the proliferation inhibitor 2′, 3′- dideoxythymidine (DDT); the movement induced by transfection with 22 miRNAs in the presence of DDT was similar to that in the primary screening (Figure 1C and Table S4).

To further confirm this result, we performed transwell migration assays in SK-HEP-1 cells transfected with the 22 miRNAs for 48 hours. Finally, 11 miRNAs (miR-134, -146b-3p, -188-3p, -525-3p, -661, -767-5p, -891a, -891b, -1244, -1247 and miR-1471) of the 22 miRNAs demonstrated consistent regulatory behavior. (Figure 1D and Table S4).

### miR-134 is inversely correlated with HCC lymphatic invasion and vascular invasion

Our previous work revealed that miR-134 is often downregulated in HCC tissues [17]. Furthermore, we investigated the correlation between the miR-134 expression level and the clinical or pathological variables in two groups (cutoff value of median), and found that miR-134 expression is inversely correlated with lymphatic invasion (p = 0.04) and vascular invasion (p = 0.04) in HCC (Table 1). However, no relationship was found between miR-134 expression and tumor size or multinodularity.

**Table 1:** Correlation of clinicopathological features with tumor miR-134 expression in HCC.

	Case(n)	miR-134 level		*P value*
		Low (<Median)	High (>Median)	
**Sex**				
Male	95	50	45	0.29
Female	18	7	11	
**Age (years)**				
≤50	71	33	38	0.27
>50	42	24	18	
**Edmondson Grading**				
I+II	33	17	16	0.8
III+IV	46	25	21	
**Tumor Size (cm)**				
<5	33	17	16	0.67
≥5	66	31	35	
**Serum AFP (ng/ml)**				
≤20	35	15	20	0.26
>20	66	36	30	
**Serum HBsAg**				
Positive	83	41	42	0.48
Negative	28	16	12	
**Liver Cirrhosis**				
Positive	52	24	28	0.45
Negative	60	32	28	
**Lymphatic Invasion**				
Positive	16	12	4	***0.04***
Negative	96	45	51	
**Distant Metastasis**				
Positive	13	8	5	0.4
Negative	100	49	51	
**Vascular Invasion**				
Positive	13	10	3	***0.04***
Negative	83	38	45	
**Multinodular Liver**				
Positive	43	24	19	0.19
Negative	52	22	30	

Note: The expression level of mature miR-134 was normalized against that of RNU6B small nuclear RNA. The P-value represents the probability from a chi-square test for expression of miR-134 between variable subgroups. AFP, alpha-fetoprotein; HBsAg, hepatitis B virus surface antigen.

### miR-134 inhibits HCC cell invasion and metastasis *in vitro* and *in vivo*

**Figure 2:**
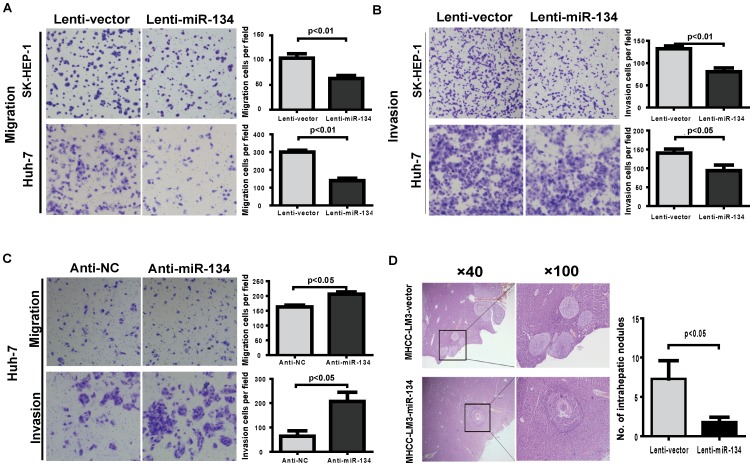
miR-134 inhibits HCC cell migration and invasion *in vitro* and *in vivo*. **A** and **B.** Transwell migration (**A**) and invasion (**B**) assays for SK-HEP-1 and Huh-7 cells stably overexpressing miR-134 or negative control (lenti-vector). Representative images are shown with the quantification of five randomly selected fields. **C.** Transwell migration and invasion assays of Huh-7 cells were performed after transfection with the miR-134 inhibitor or negative control. Differences between two groups were analyzed by the Mann-Whitney test. **D.** H & E staining of liver isolated from mice that received orthotopic injections of miR-134-transduced or vector-infected LM3 cells at 8 weeks after transplantation. Intrahepatic nodules were counted and analyzed using the Mann-Whitney test (n = 10 mice per group).

To determine whether the enhanced expression of miR-134 inhibits HCC cell invasion and metastasis, we first measured the expression of miR-134 in various HCC cell lines and constructed three miR-134-overexpressing stable cell lines by lentiviral transduction (Figure S1A in File S1). Cell proliferation and colony formation assays revealed that the ectopic expression of miR-134 had no obvious effects on HCC cell proliferation *in vitro* (Figure S1B–D in File S1), while transwell experiments showed that the cells with stable miR-134-overexpression, which were derived from SK-HEP-1, MHCC-LM3 and Huh-7 cells, exhibited a lower migratory and invasive potential than the control cells (Figure 2A, 2B and Figure S2 in File S1). In contrast, the suppression of endogenous miR-134 by its specific inhibitor enhanced the migratory and invasive abilities in Huh-7 cells (Figure 2C). To further explore the role of miR-134 in tumor invasion and metastasis *in vivo*, MHCC-LM3 cells, which possess a relatively strong *in vivo* metastatic capacity, were orthotopically transplanted into the livers of nude mice. The number of intrahepatic nodules in the liver was dramatically decreased in the miR-134 groups compared to the vector controls after *in situ* growth for 8 weeks (Figure 2D). Together, these findings suggest that miR-134 is a negative regulator of metastasis in HCC.

### miR-134 impacts ITGB1 expression by directly binding to its 3′-UTR

**Figure 3:**
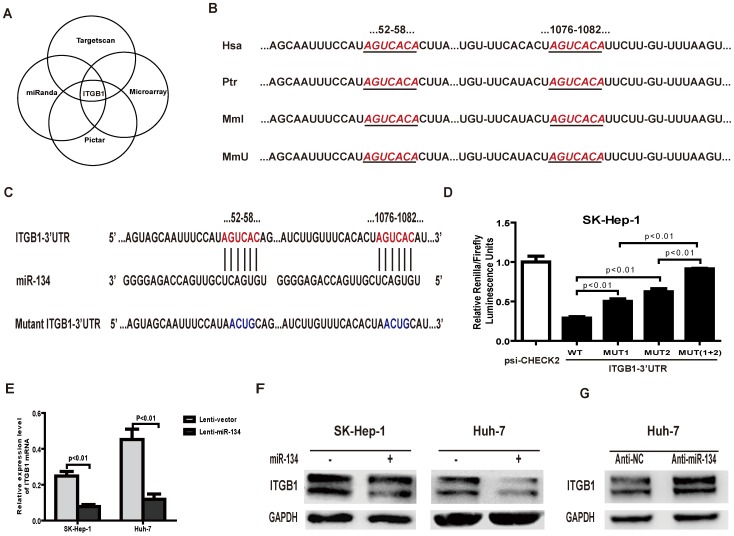
miR-134 downregulates ITGB1 by directly targeting its 3′UTR. **A.** Schema of the candidate genes identified by four independent approaches. **B.** Putative binding sequences of ITGB1 for miR-134 in the human (Hsa), chimpanzee (Ptr), rhesus (Mml) and mouse (Mmu). The ITGB1 seed sequences are highlighted by ruby italic letters. **C.** Putative miR-134-binding sites in the wild-type ITGB1 3′-UTR (highlighted by ruby letters) or 3′-UTR segments containing mutant (highlighted by blue letters) binding sites are shown. **D.** Relative luciferase activity assays for constructs containing luciferase and wild-type or mutant ITGB1 3′-UTRs were performed after cotransfection with a miR-134 mimic or negative control. The *Renilla* luciferase vector was cotransfected as an internal control. Data were analyzed by one-way ANOVA (p<0.01), and differences between two groups were assessed by the Bonferroni post-test. **E.** The mRNA expression levels of ITGB1 in SK-HEP-1 and Huh-7 cells overexpressing miR-134 or negative control. Differences between two groups were analyzed by the Mann-Whitney test. **F.** The protein levels of ITGB1 in SK-HEP-1 and Huh-7 cells infected with miR-134 or control lentivirus. GAPDH served as an internal control. **G.** Protein levels of ITGB1 in Huh-7 cells after transfection with the miR-134 inhibitor or inhibitor control. GAPDH served as an internal control.

Generally, miRNAs exert their function by binding to the 3′-UTR of their target genes, blocking subsequent protein expression. Integrin beta 1 (ITGB1) and cadherin 9 (CDH9) were predicted to be the target genes of miR-134 by combining three target-predicting algorithms (PicTar, www.pictar.org, TargetScan, www.targetscan.org and miRanda, www.microrna.org). In addition, we performed an RNA microarray assay in SK-HEP-1 cells, and the results showed that the ITGB1 mRNA expression level decreased by 66% in miR-134 overexpressing cells compared with control cells (the expression ratio was 0.341524), whereas CDH9 mRNA expression levels remained relatively stable. These results indicate that ITGB1 is a putative target gene of miR-134 (Figure 3A).

Next, analysis of the 3′-UTR sequence of ITGB1 using TargetScan revealed two possible binding sites of miR-134, both of which are highly conserved in humans, chimpanzees, rhesus monkeys and mice (Figure 3B). To further determine the direct binding of miR-134 to the 3′-UTR, the fragments of the ITPG1 3′-UTR (either wild-type or mutant, Figure 3C) were constructed and inserted downstream of the stop codon of Renilla luciferase in the psiCHECK2 vector. MiR-134 was cotransfected with different 3′-UTR constructs into SK-HEP-1 cells. Compared to the control, the relative luciferase activity of wild-type 3′-UTR of ITGB1 was sharply decreased by miR-134, whereas the relative luciferase activity of the 3′-UTR construct containing two mutant binding sites was not altered. In addition, our results indicate that the two binding sites act synergically in miR-134 regulation because single mutation of these two binding sites only partially restored the luciferase activity (Figure 3D). Similar results were observed when different 3′-UTRs constructed with psiCHECK2 were transfected into Huh-7 cells with the miR-134 mimic (Figure S3 in File S1). Furthermore, overexpression of miR-134 remarkably decreased the endogenous mRNA and protein levels of ITGB1 (Figure 3E, 3F). Conversely, inhibition of miR-134 increased the expression of ITGB1 in Huh-7 cells (Figure 3G). Collectively, these data provide further evidence that ITGB1 is a direct target of miR-134.

### Restoration of ITGB1 abrogates miR-134-repressed HCC cell migration and invasion

**Figure 4:**
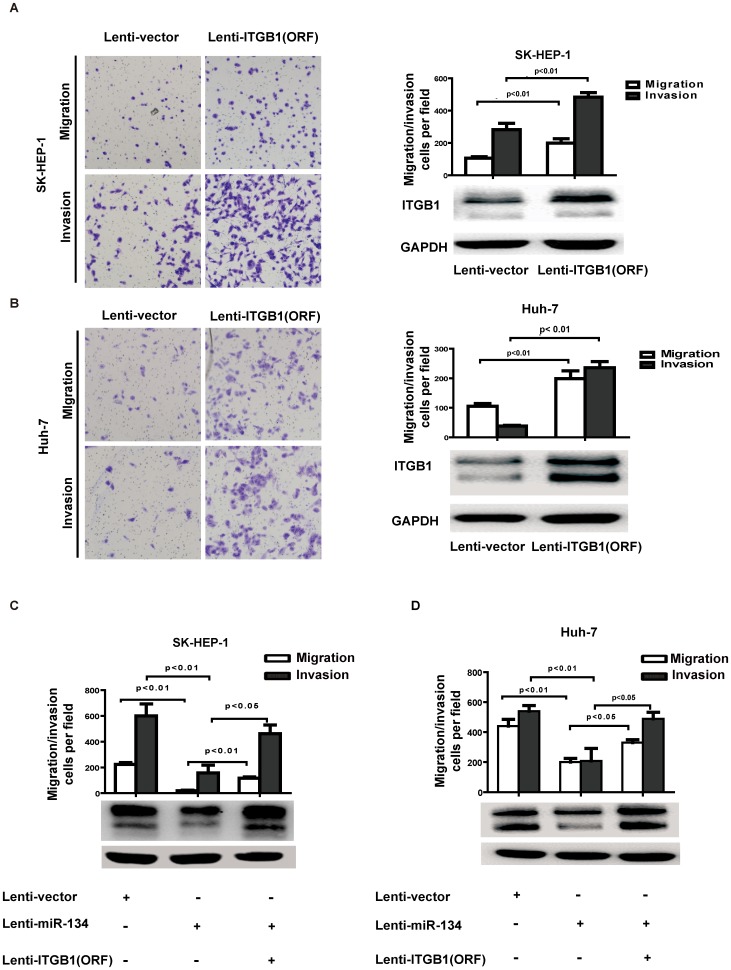
Restoration of ITGB1 expression rescues miR-134-induced suppression of HCC migration and invasion. **A** and **B.** Transwell migration assays of (A) SK-HEP-1 and (B) Huh-7 cells stably overexpressing ITGB1. Representative images are shown with the quantification of five randomly selected fields. The bars represent the SEM of three independent experiments. Differences between two groups were analyzed by the Mann-Whitney test. Western blot analysis revealed that the protein expression of ITGB1 increased after infection with the PLVX-ITGB1 (ORF) virus. **C.** Transwell migration and invasion assays for SK-HEP-1-vector or SK-HEP-1- miR-134 cells were performed after infection with ITGB1 (ORF) or the vector control lentivirus. Transwell migration and invasion data were analyzed by a one-way ANOVA (p<0.01, p<0.01 respectively); differences between two groups were assessed by the Bonferroni post-test. ITGB1 protein levels were determined by Western blot analysis. **D.** Transwell migration and invasion assays for Huh-7-vector or Huh-7-miR-134 cells were performed after infection with ITGB1 (ORF) or vector control lentivirus. ITGB1 protein levels were determined by Western blot analysis. Transwell migration and invasion data were analyzed by a one-way ANOVA (p<0.01, p<0.01 respectively); differences between two groups were assessed by the Bonferroni post-test.

Previous studies showed that the mRNA level of ITGB1 was upregulated in HCC samples compared to the adjacent normal liver tissue [20]. To explore whether ITGB1 contributes to the suppressive effect of miR-134, the complete ORF of ITGB1 without its 3′-UTR, which resulted in the constitutive expression of ITGB1 resistant to miR-134 inhibition, was introduced into SK-HEP-1 and Huh-7 cells via lentiviral transduction. Remarkably, overexpression of ITGB1 strongly increased SK-HEP-1 and Huh-7 cell migration and invasion (Figure 4A and 4B). We further observed that ectopic ITGB1-ORF without 3′UTR in miR-134-transduced SK-HEP-1 and Hun-7 cells abrogated the inhibitory effect of miR-134 on HCC migration and invasion (Figure 4C and 4D). Together, these findings provide evidence that miR-134 suppresses HCC cell invasion and metastasis by directly targeting ITGB1.

### Overexpression of miR-134 represses HCC cell stress fiber formation and cell adhesion

**Figure 5:**
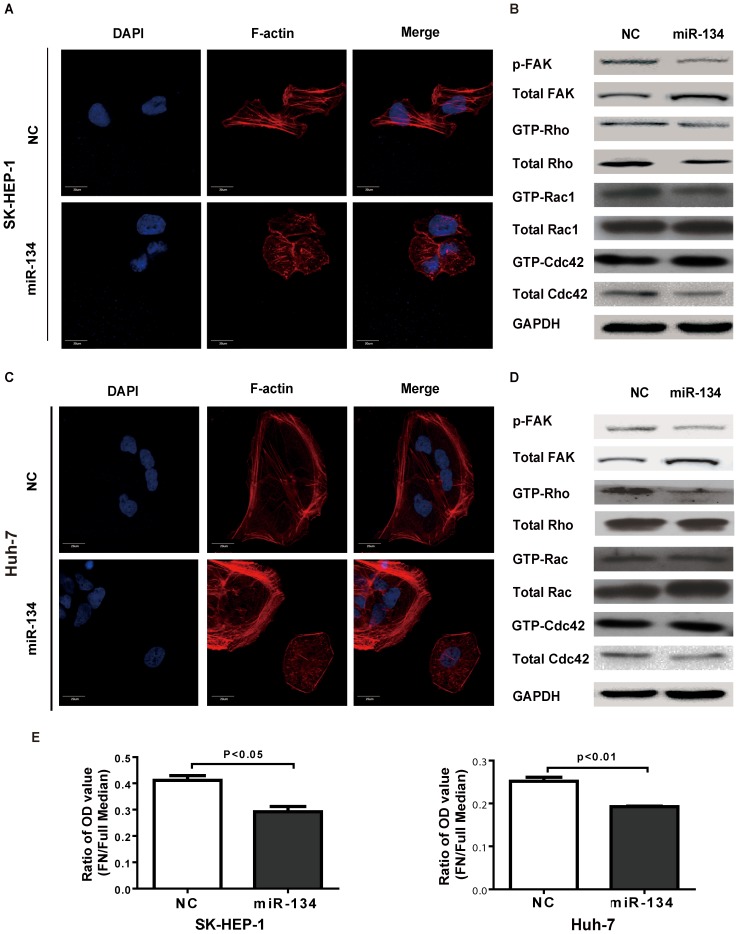
Ectopic expression of miR-134 impairs the stress fiber network in HCC cells. **A.** and **C.** Ectopic expression of miR-134 disrupted the stress fiber network in SK-HEP-1 (**A**) and Huh-7 cells (**C**). Stress fibers (polymerized actin) and actin filaments were detected by phalloidin staining. DAPI staining was used to detect nuclei. Scale bar: 20 µm. **B.** and **D.** The protein levels of FAK phosphorylation and activity of Rho/Rac1/Cdc42 GTPases in SK-HEP-1 (**B**) and Huh-7 (**D**) cells. Samples for the Western blot were analyzed on a single gel. **E.** Cellular adhesion assays were performed in SK-HEP-1 and Huh-7 cells. The results are representative of at least three independent experiments. Differences between two groups were analyzed by the Mann-Whitney test. NC: negative control, FN: fibronectin.

To further study the role of miR-134 in cell motility, we examined the status of stress fiber formation and polymerized actin in SK-HEP-1 and Huh-7 cells with miR-134 overexpression. Using phalloidin staining, we found that the maturation of stress fibers was suppressed in cells transfected with the miR-134 mimic (Figure 5A and 5C). However, miR-134 did not affect the consistency of vimentin or tubulin, which implies that miR-134 does not interfere the formation of intermediate filaments (Figure S4 in File S1). Previous work has shown that phosphorylated focal adhesion kinase (FAK) activates RhoA and drives the formation of stress fibers and the maturation of cell adhesion [21]; thus, we determined the status of the FAK signaling pathway, including the phosphorylation of FAK and downstream activation of Rho, Rac1 and Cdc42. Western blotting showed that miR-134 attenuated FAK phosphorylation and suppressed the activation of RhoA in SK-HEP-1 and Huh-7 cells (Figure 5B and 5D). Moreover, cell adhesion assays also revealed that miR-134 inhibited cell-matrix adhesion in SK-HEP-1 and Huh-7 cells (Figure 5E).

**Figure 6:**
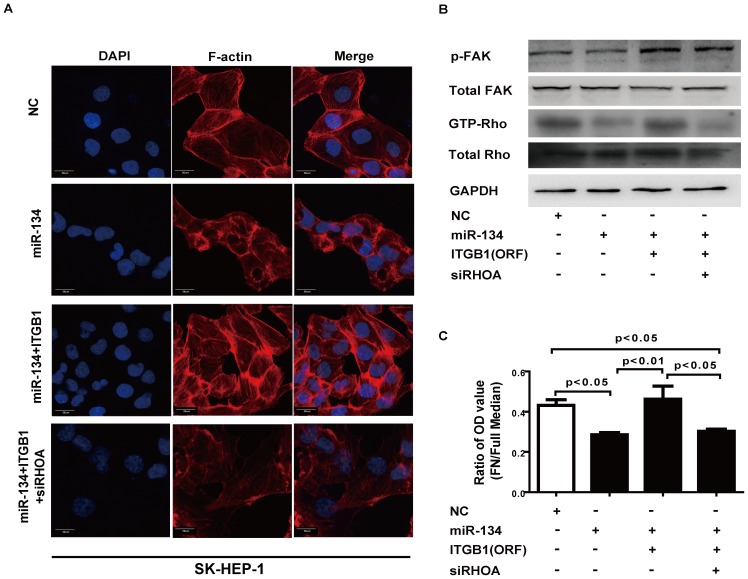
ITGB1 is involved in the miR-134-induced inhibition of cell stress fiber formation and cell adhesion. **A.** Ectopic expression of miR-134 disrupted the stress fiber network in SK-HEP-1 cells, and this effect was reversed by ITGB1 transfection. Stress fiber formation was repressed by transfecting siRhoA into SK-HEP-1-ITGB1 cells. F-actin was used as the stress fiber marker. DAPI staining was used to detect nuclei. Scale bar: 20 µm. **B.** FAK protein phosphorylation and the protein levels of the Rho GTPases in SK-HEP-1-ITGB1 cells were analyzed after transfection with miR-134 and siRhoA. **C.** The cellular adhesion assay was performed in SK-HEP-1 cells. The results are representative of at least three independent experiments, and data were analyzed by a one-way ANOVA (p<0.05); differences between two groups were assessed by the Bonferroni post-test. NC: negative control, FN: fibronectin.

In addition, ectopic ITGB1 abrogated miR-134-suppressed cell stress fiber formation. Nevertheless, this abrogation was reversed by siRNA knockdown of RhoA (Figure 6A, 6B, Figure S5A and S5B in File S1). Similarly, reintroduction of ITGB1 rescued miR-134-suppressed cell adhesion, and this effect was abrogated by knockdown of RhoA (Figure 6C and Figure S5C in File S1). Taken together, these data suggest that ITGB1 plays a pivotal role in miR-134-mediated cell stress fiber formation and cell adhesion.

## Discussion

Previous studies have shown that time-lapse imaging of living cells is versatile for investigating cell mobility. This technique allowed us to investigate many phenotypes and morphologies associated with cell movement and mobility [22], [23]. In this study, we used time-lapse video microscopy to observe cell movement in the scratch-wound assay, validated the results using the transwell migration assay, and identified the migration-suppressing miRNA (miR-134) and migration-facilitating miRNAs (miR-1247, miR-1244, miR-146b-3p, miR-1471, miR-188-3p, miR-661, miR-891a, miR-891b and miR-767-5P) in SK-HEP-1 cells.

miR-134 is located on chromosome 14q32.31, which is frequently lost in gastrointestinal stromal tumors (GISTs) [24], and its expression is often decreased in GIST, osteosarcoma, glioma and HCC [17], [24], [25], [26]. Boominathan reported that TA-p73 may regulate the post-transcriptional processing of miR-134 by increasing Ago1/2 activity, thereby repressing cancer stem cell proliferation [27]. However, the regulation of miR-134 expression in HCC remains to be elucidated. Moreover, low expression of miR-134 is significantly associated with shorter disease-free survival or tumor progression in GIST. Additionally, miR-134 inhibits cell migration and EMT in A549 cells [28], [29]. Our previous work also demonstrated that miR-134 is often downregulated in HCC tissues, and its expression level is reversely correlated with HCC lymphatic invasion and vascular invasion. We also verified that ectopic expression of miR-134 suppresses HCC cell invasion and metastasis both *in vitro* and *in vivo*.

The present work shows that miR-134 participates in the integrin-signaling pathway by directly regulating ITGB1 expression. ITGB1, which is a subunit of a family of heterodimeric transmembrane receptors, can combine with different integrin α subunits to form heterodimers and bind a large number of extracellular ligands. For example, α1β1, α2β1, α10β1 and α11β1 are collagen-binding integrins; α3β1, α6β1 and α7β1 bind to laminin and α4β1, α5β1, α8β1 and αvβ1 bind to fibronectin [30]. In many cancer types, integrin beta 1 promotes cancer cell proliferation and survival and regulates cell focal adhesion and tumor metastasis [31], [32], [33], [34]. Masumoto reported that a monoclonal antibody against ITGB1 blocked HCC cell invasion [35], and Mizuno found that overexpression of α3β1 integrins enhanced HepG2 cell migration and invasion [36], while inhibition of α3β1 integrins suppressed SMMC-7721 cell migration [37]. Our study showed that ITGB1 promotes HCC cell migration and invasion and rescues miR-134-induced repression of cell migration and invasion. In addition, ITGB1 mRNA was upregulated in HCC tissues compared with the adjacent normal liver tissue [20].

When extracellular ligands bind to integrins, their cytoplasmic domains connect to intracellular signaling molecules to activate an immediate signal transduction pathway or crosstalk with growth factor receptors. Then, FAK, the key molecule of the integrin-signaling pathway, is recruited to the integrin C-terminal domain and autophosphorylated at tyrosine397, thereby creating a binding site for the Src-homology 2 (SH2) domain of Src. The active FAK-Src complex then promotes the activation of the small Rho family members, Rac1, Cdc42 and RhoA [21]. RhoA is a crucial molecule in cell cytoskeleton remodeling and actin reorganization because it activates myosin II through Rho-kinase (ROCK) and contributes to actin polymerization in the formation of stress fibers [38]. In addition to the inhibitory effect of miR-134 on ITGB1 expression, our data demonstrated that miR-134 affects FAK phosphorylation and RhoA activation in HCC cells. GTP-Rho is decreased and the formation of stress fibers is inhibited by overexpression of miR-134. Overexpression of ITGB1 could reverse the inhibitory effect of miR-134 by increasing of GTP-Rho. When we knockdown RhoA, GTP-Rho decreases and the formation of stress fibers is suppressed.

Cell migration is frequently composed of the cycles consisting of protrusion at the front, the formation and stabilization of adhesions at the front and then the retraction and destabilization of adhesions at the rear [38]. Protrusion may be mediated by a variety of distinct structures, including lamellipodia, filopodia and stress fibers. Rac1 stimulates lamellipodium formation, Cdc42 induces filopodium formation and RhoA induces the formation of stress fibers [39]. As miR-134 could reduce ITGB1 expression and the consequent FAK phosphorylation and RhoA activation, it is not surprising to find that miR-134 inhibits cell adhesion and migration in HCC.

In conclusion, we identified potential tumor metastasis-regulating miRNAs through a cell migration assay. Our results show that miR-134 significantly inhibits HCC cell invasion and metastasis *in vitro* and *in vivo* and further highlights the critical role of ITGB1 and its downstream pathway in miR-134-mediated inhibition of metastasis in HCC. This work suggests that miR-134 could be a novel therapeutic target for the treatment of HCC.

## Supporting Information

Table S1 **List of human miRNAs tested in the study.**
(XLS)

Table S2 **Primer sequences for real time PCR and constructs.**
(XLS)

Table S3 **Clinical information of human hepatocellular carcinoma samples used in the study.**
(XLS)

Table S4 **List of miRNAs capable of regulating HCC cell migration.**
(XLS)

File S1 **Supporting information on results obtained, containing Figure S1, S2, S3, S4 and S5.** Figure S1, Overexpression of miR-134 has no effect on HCC cell growth. **A.** the relative expression levels of miR-134 in HCC cells. The expression levels of mature miR-134 are normalized by U6 small nuclear RNA. The CCK-8 assays of SK-HEP-1 (**B**) and Huh-7 (**C**) cells were performed after infected with lentivirus expressing miR-134 or the control vector. **D.** The colony formation assay for SK-HEP-1 cells infected with lentivirus expressing miR-134 or the control vector. A total of 500 cells per well were seeded and cultivated for 2 weeks. The colonies were fixed and stained in a dye solution containing 0.1% crystal violet and 20% methanol. Figure S2, Overexpression of miR-134 inhibits MHCC-LM3 cell migration and invasion. Transwell migration assays of MHCC-LM3 cells were performed after infection with lentivirus expressing miR-134 or the control vector. For the migration assay, 10^5^ cells were placed into the top chamber of the insert and cultured for 28 hours. For the invasion assay, 2*10^5^ cells were added to the upper chamber of the insert which had previously been coated with 40 µl of Matrigel and cultured for 48 hours. Cells that had migrated through the pore were fixed and stained with a mixture of 20% methanol and 0.1% crystal violet for 0.5 hour. Differences between two groups were analyzed by the Mann-Whitney test. Figure S3, miR-134 directly targets 3′-UTR of ITGB1 The psiCHECK2 vector, psiCHECK2-wild-ITGB1-3′UTR or psiCHECK2-mutant- ITGB1-3′UTR was transfected into Huh-7 cells with miR-134 mimic or negative control. Data were analyzed by one-way ANOVA (p<0.01), and differences between two groups were assessed by the Bonferroni post-test. Figure S4, Overexpression of miR-134 has no effect on microtubule and intermediate filament in HCC cells. Microtubules were demonstrated by α-tubulin staining using α-tubulin mAb in SK-HEP-1 (**A**) and Huh-7 (**B**) cells. DAPI staining is used to detect nuclei. Scale bar: 20 µm. Intermediate filaments were demonstrated by Vimentin using Vimentin mAb in SK-HEP-1 (**C**) and Huh-7 (**D**) cells. DAPI staining is used to detect nuclei. Scale bar: 20 µm. Figure S5, ITGB1 contribute to the inhibition of cell stress fiber formation and cell adhesion by miR-134. **A.** Ectopic expression of miR-134 disrupted the stress fiber network in Huh-7 cells, and this effect was reversed by ITGB1 transfection. Stress fiber formation was repressed by transfecting siRhoA into Huh-7-ITGB1 cells. F-actin was used as the stress fiber marker. DAPI staining was used to detect nuclei. **B.** FAK protein phosphorylation and the protein levels of the Rho GTPases in Huh-7-ITGB1 cells were analyzed after transfection with miR-134 and siRhoA. **C.** The cellular adhesion assay was performed in Huh-7 cells. The results were representative of at least three independent experiments, and data were analyzed by a one-way ANOVA (p<0.01); differences between two groups were assessed by the Bonferroni post-test.
(DOC)

Video S1 **Cell migration after treatment with negative control.**
(MP4)

Video S2 **Cell migration after treatment with miR-891a.**
(MP4)

Video S3 **Cell migration after treatment with miR-134.**
(MP4)
