# Supplementary figures and images for: Genome-Wide Screening Identified That miR-134 Acts as a Metastasis Suppressor by Targeting Integrin β1 in Hepatocellular Carcinoma
Source: PLoS One. 2014 Feb 3;9(2):e87665. doi: 10.1371/journal.pone.0087665 (PMC3912066; doi:10.1371/journal.pone.0087665)

**Supplementary Figures**

**Figure S1**

**
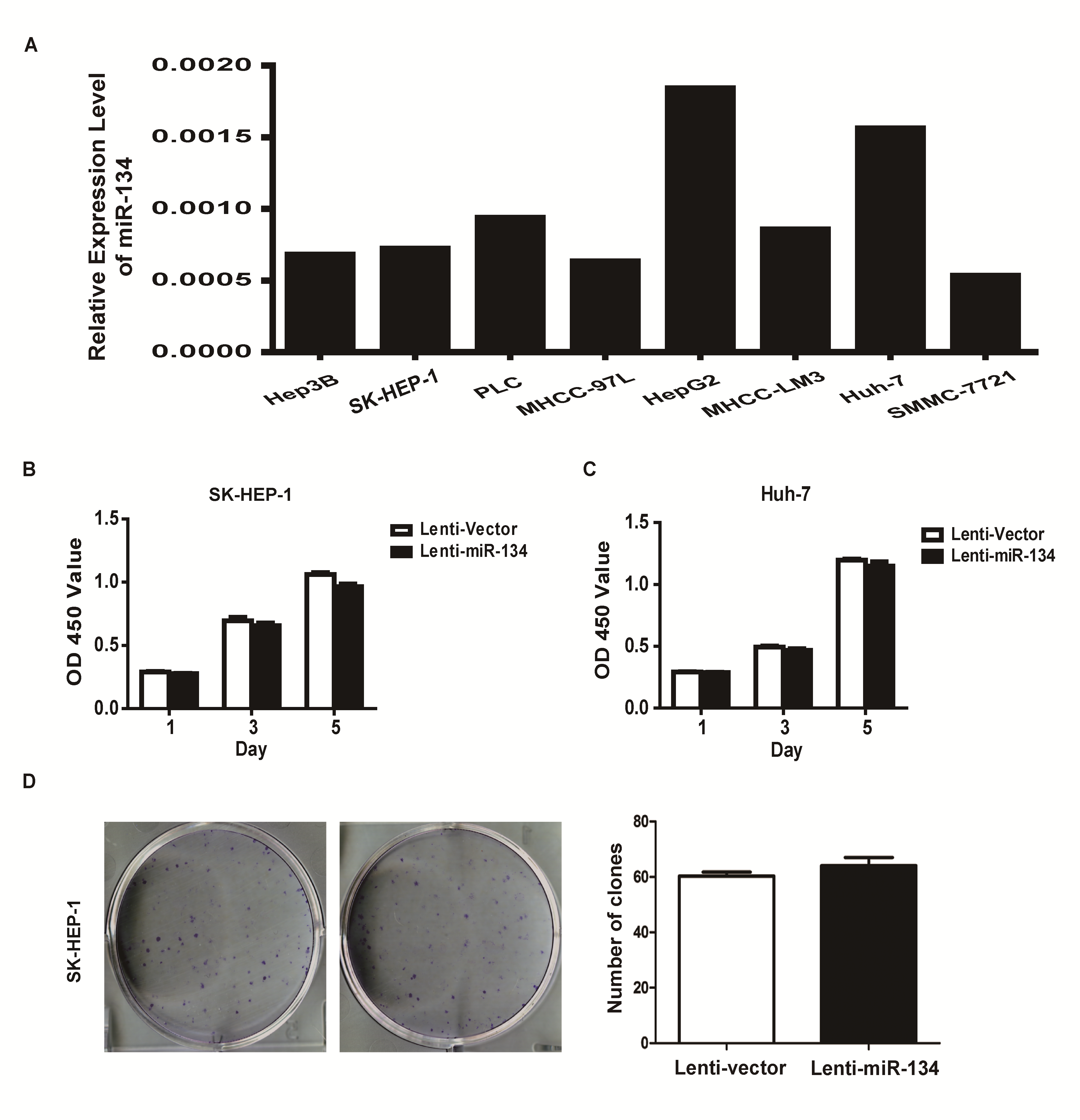
**

**Figure S2**


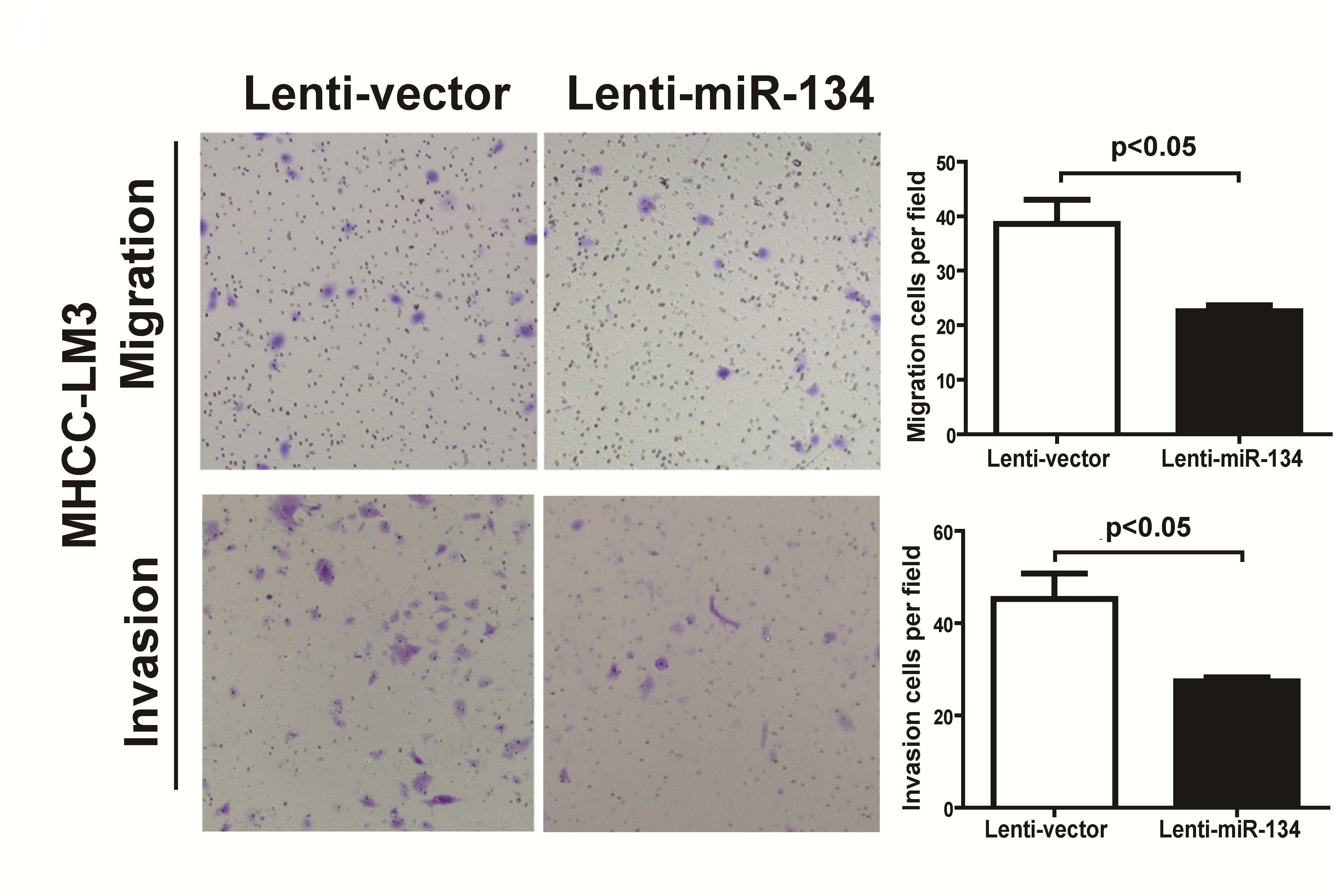


**Figure S3**

**
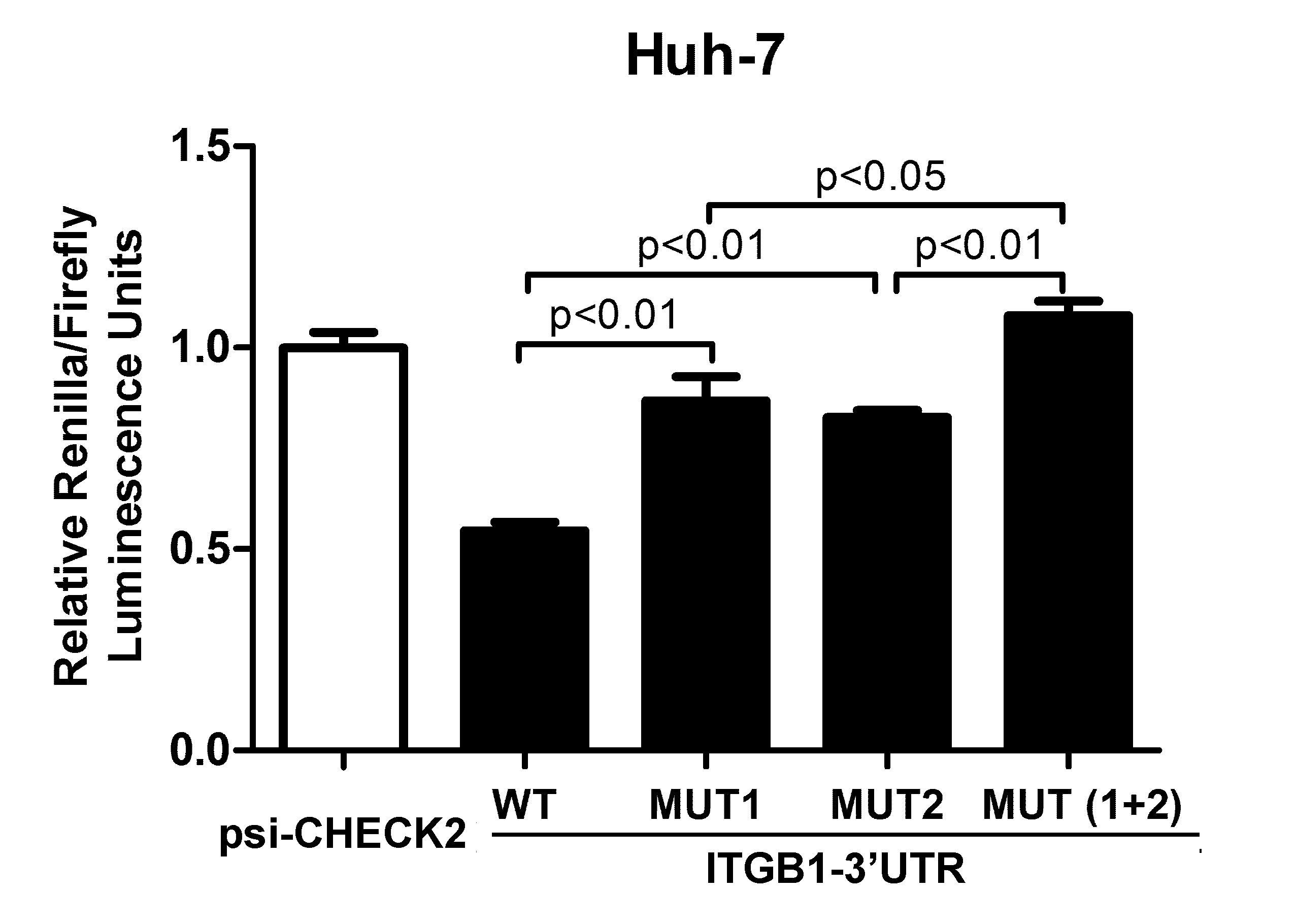
**

**Figure S4**


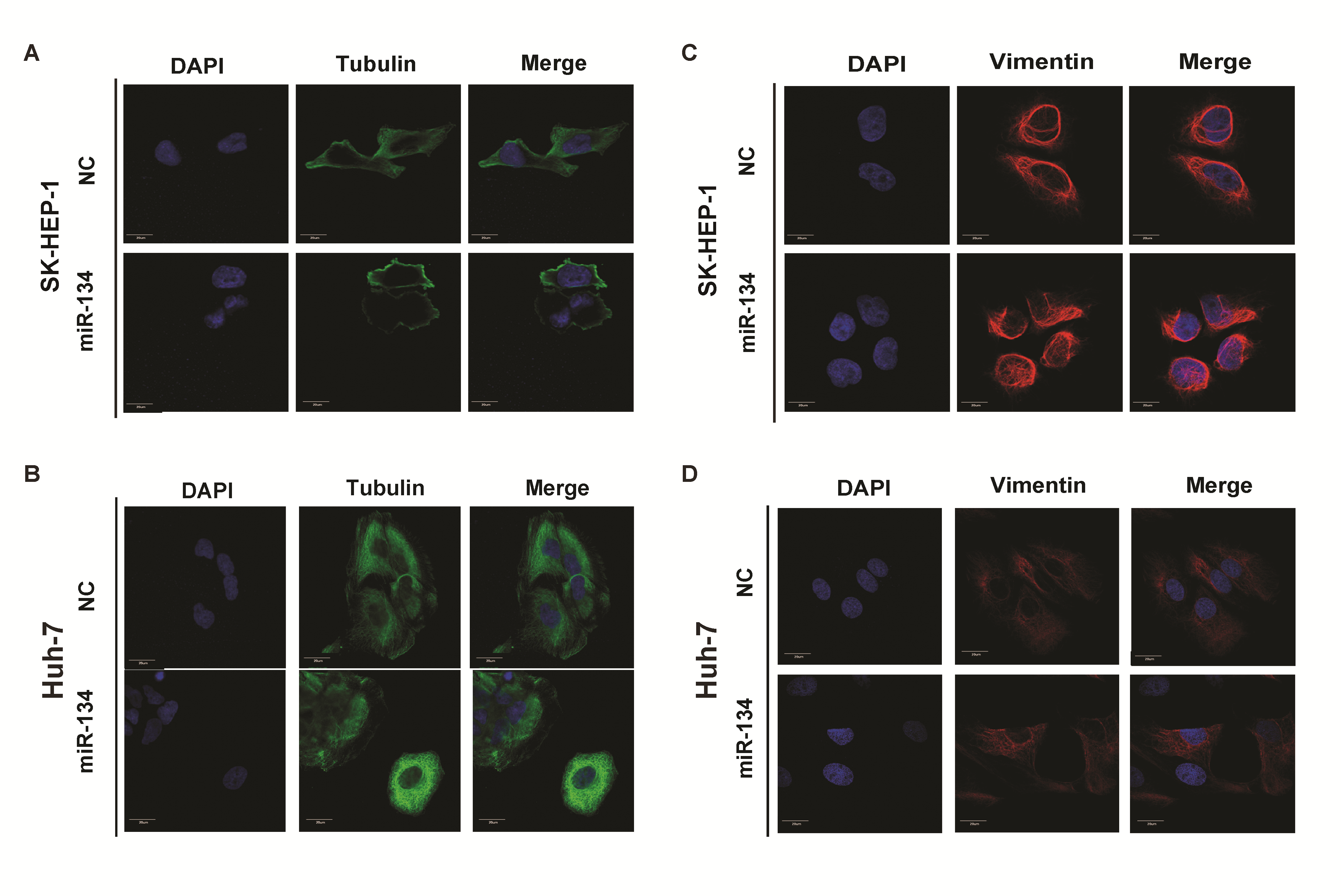


**Figure S5**

**
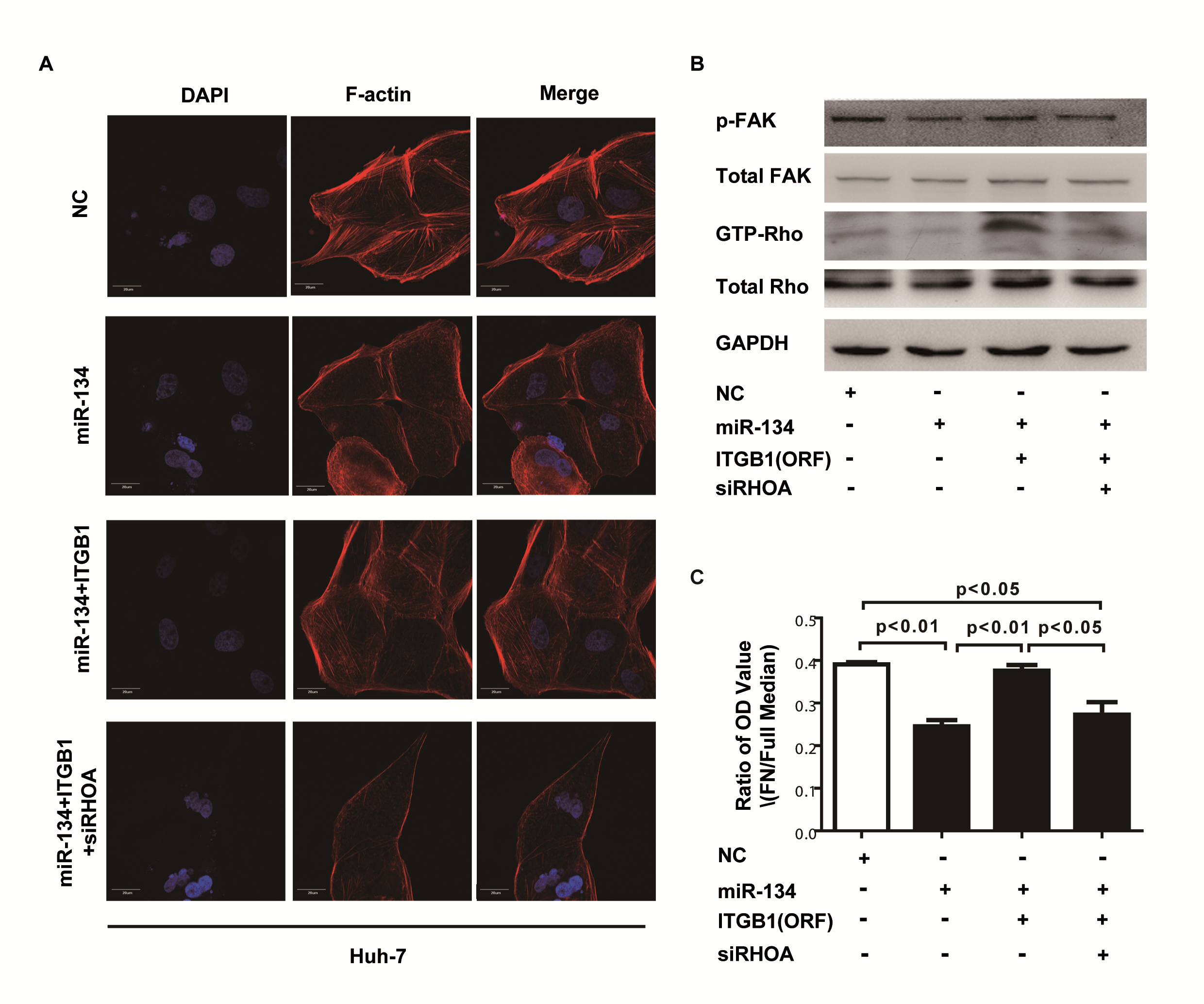
**

Supplement: File S1 — Supporting information on results obtained, containing Figure S1, S2, S3, S4 and S5. Figure S1, Overexpression of miR-134 has no effect on HCC cell growth. A. the relative expression levels of miR-134 in HCC cells. The expression levels of mature miR-134 are normalized by U6 small nuclear RNA. The CCK-8 assays of SK-HEP-1 (B) and Huh-7 (C) cells were performed after infected with lentivirus expressing miR-134 or the control vector. D. The colony formation assay for SK-HEP-1 cells infected with lentivirus expressing miR-134 or the control vector. A total of 500 cells per well were seeded and cultivated for 2 weeks. The colonies were fixed and stained in a dye solution containing 0.1% crystal violet and 20% methanol. Figure S2, Overexpression of miR-134 inhibits MHCC-LM3 cell migration and invasion. Transwell migration assays of MHCC-LM3 cells were performed after infection with lentivirus expressing miR-134 or the control vector. For the migration assay, 105 cells were placed into the top chamber of the insert and cultured for 28 hours. For the invasion assay, 2*105 cells were added to the upper chamber of the insert which had previously been coated with 40 µl of Matrigel and cultured for 48 hours. Cells that had migrated through the pore were fixed and stained with a mixture of 20% methanol and 0.1% crystal violet for 0.5 hour. Differences between two groups were analyzed by the Mann-Whitney test. Figure S3, miR-134 directly targets 3′-UTR of ITGB1 The psiCHECK2 vector, psiCHECK2-wild-ITGB1-3′UTR or psiCHECK2-mutant- ITGB1-3′UTR was transfected into Huh-7 cells with miR-134 mimic or negative control. Data were analyzed by one-way ANOVA (p<0.01), and differences between two groups were assessed by the Bonferroni post-test. Figure S4, Overexpression of miR-134 has no effect on microtubule and intermediate filament in HCC cells. Microtubules were demonstrated by α-tubulin staining using α-tubulin mAb in SK-HEP-1 (A) and Huh-7 (B) cells. DAPI staining is used to detect [file pone.0087665.s005.doc]
